# Review: immunoassays in DNA damage and instability detection

**DOI:** 10.1007/s00018-019-03239-6

**Published:** 2019-07-24

**Authors:** Karolina Boguszewska, Michał Szewczuk, Sandra Urbaniak, Bolesław T. Karwowski

**Affiliations:** DNA Damage Laboratory of Food Science Department, Faculty of Pharmacy, Medical University of Lodz, ul. Muszynskiego 1, 90-151 Lodz, Poland

**Keywords:** DNA damage, Immunoassay, Tyramide signal amplification (TSA), Time resolved amplified cryptate emission (TRACE), Surface plasmon resonance (SPR), Quantum dots (QDs)

## Abstract

The review includes information on the current state of knowledge of immunometric methods with emphasis on the possibility of deoxyribonucleic acid (DNA) damage detection. Beginning with basic immunoassay enzyme-linked immunosorbent assay (ELISA), this review describes methods such as tyramide signal amplification (TSA), enhanced polymer one-step staining (EPOS), and time resolved amplified cryptate emission (TRACE) as improvements of ELISA’s developed over time to obtain more accurate results. In the second part of the review, surface plasmon resonance (SPR) and quantum dots (QDs) are presented as the newest outlooks in the context of immunoanalysis of biological material and molecular studies. The aim of this review is to briefly present immunoassays with emphasis on DNA damage detection; therefore, the types of methods are listed and described, types of signal indicators, basic definitions such as antigen and antibody are given. Every method is considered with an exemplary application focusing on DNA studies, DNA damage and instability detection.

## Introduction

Development of immunometric assays is highly relevant for the progress in immunochemistry and other fields. Type and sensitivity of a specific method translate into quality and precision of obtained analytical results. Accuracy of results is highly important in DNA damage experiments due to the fact that small differences in results may carry great meaning and/or indicate false positive results. Research is being carried out worldwide to develop new or refine current immunometric methods. In this review, the basics of immunoassays are described together with new methods that are useful in the study of small particles such as nucleotides.

Constant process of damage and repair takes place in the cells. DNA damage is caused by a number of factors including metabolic processes, where reactive oxygen species (ROS), reactive nitrogen species, lipid peroxidation products, and others may be released. Oxidative damage which occurs naturally happens for minimum 10,000 times per day in *Homo sapiens* organism. Nowadays, environmental factors including food contaminants have a great impact on the level of DNA damage emerging in humans. UV light (ultraviolet light), ionizing radiation, and genotoxic chemicals such as vinyl chloride, hydrogen peroxide or polycyclic aromatic hydrocarbons (found in smoke) are only a few factors which may interact with our bodies at the molecular level and, in many cases, lead to serious diseases. More than 70 types of DNA damage can be distinguished, including oxidative damage (e.g., 8-hydroxy-2′-deoxyguanosine (8-oxo-dG)), depurinations, depyrimidinations, single-strand breaks (SSB), double-strand breaks (DSB), cytosine deaminations, O6-methylguanines, and others [1].

Common strategies to detect DNA damage comprise PCR (polymerase chain reaction), comet assay, TUNEL assay (terminal deoxyribonucleotidyl transferase-mediated deoxyuridine triphosphate nick end labeling), FISH (fluorescence in situ hybridization), FCM (flow cytometry), halo assay (propidium iodide (PI) labeling), annexin V labeling, immunological and immunohistochemical assays such as ELISA, radioimmunoassays (RIA), gel electrophoresis, chromatography techniques, and others [2–4].

Mapping DNA damage with *PCR* is widely adapted, as the DNA breakage stops the amplification during the procedure. Even in the case of such a standard technique, improvement is being made—the development of immuno-coupled PCR (ICPCR) allows to analyze thymine dimers at the gene level in human DNA through combining amplification with biotinylated DNA which is bound to antigen–antibody complex and SINE (short interspersed DNA element) allows to detect damage and repair levels of DNA including adducts of cisplatin to DNA or modifications induced by UV-B light [5–8]. *Comet assay* (single cell electrophoresis) is another standard technique which detects overall damage of single cells caused mainly by radiation; SSBs, DSBs, oxidative DNA damage or even pyrimidine dimers may be observed within the population of cells. Improvements being made, the comet–FISH method allows to analyze distribution of different types of DNA damage within the genome due to labeling sequence of interest [9]. *FISH* is a technique using fluorescently labeled DNA probes thanks to which it is possible to locate specific DNA sequences on chromosomes. For patients with breast cancer, this method allows to determine mutations in HER-2 (human epidermal growth factor receptor 2). Another procedure was developed for patients with chronic myelogenous leukemia (CML)—dual-fusion and interphase dual-color FISH to assess patient after stem cell transplantation [5]. On the other hand, peptide nucleic acid probes (PNAs) are used in Q-FISH (quantitative fluorescence in situ hybridization). PNAs, as synthetic oligonucleotide probes, have a higher affinity comparing to RNA or DNA; therefore, they are used as probes to quantify sequences of interest on chromosomes. The most common application of the Q-FISH method is telomere length studies and DNA double-strand breaks that are associated with cancer and aging [10, 11].

Assays based on antibody interactions show distinct benefits in the context of the DNA damage and instability detection such as sensitivity, selectivity, adaptability, and possibility of testing samples obtained from cells and tissues. In this review, we focus and describe in greater detail the group of immunoassays and other methods which employ principles and/or their elements.

## DNA damage

DNA is the most important molecule in every living cell as it stores genetic information. Changes in bases or their complementarity (damage of nucleobases and/or sugar, covalent bonds between bases) may lead to mutations and have impact on overall health of the organism. DNA damage is categorized as exogenous or endogenous. Exogenous DNA damage is caused by many extracellular factors such as ionizing radiation (X-rays, gamma, beta, alfa), UV radiation (UV-A: 380–320 nm, UV-B: 320–290 nm, UV-C: 290–190 nm), environmental pollution, chemotherapeutics, smoking, etc. [12]. These factors impact the body on molecular level causing formation of, e.g., SSBs (single-strand breaks), DSBs (double-strand breaks), and 8-oxo-guanine for ionizing radiation, CPDs (cyclobutane pyrimidine dimers) and 6–4PPs (pyrimidine (6–4) pyrimidone photoproducts) for UV radiation. Different group of lesions are tandem base modifications including DNA–protein cross-links, purine 5′8-cyclonucleosides, and interstrand cross-links. Endogenous DNA damage include replication errors, base mismatches, DNA–topoisomerase complexes, base deamination/oxidation/methylation, and AP (apurinic/apyrimidinic) sites [13].

Worth mentioning is one of the main causes of the DNA damage—hydroxyl radical (^·^OH) which interacts with nucleotides through its addition to the double bond in bases or the abstraction of a proton from a sugar or nucleobase. ^·^OH is generated through water radiolysis (indirect effect of radiation) or Fenton’s reactions (including metal ions, e.g., Fe^2+^) and reacts mainly at the site of its generation [12]. Studies have shown that ^·^OH prefers to add to C5 in thymine and cytosine or abstract hydrogen atom in methyl group. In case of guanine, ^·^OH adds to C8 and abstract H-atom from 2-amino group. Adenine addition of ^·^OH takes place also on C8; however, oxidation products of adenine are not commonly found [14, 15]. The main oxidation products present in cellular DNA are listed in Table 1.

**Table 1:** Types of oxidative modifications of nucleobases present in cellular DNA [14, 15]

Target	Product in DNA
Thymine	5,6-dihydroxy-5,6-dihydrothymine (thymine 5,6-glycols, Thy–Gly)
	5-hydroxy-5-methylhydantoin (Hyd–Thy)
	5-hydroxymethyluracil (5-HmUra)
	5-formyluracil (5-FoUra)
Cytosine	5-hydroxycytosine (5-OHCyt) 5,6-dihydroxy-5,6-dihydrouracil (uracil 5,6-glycols, Ura–Gly)
	5-hydroxyhydantoin (Hyd–Ura)
	1-carbamoyl-3,4-dihydroxy-2-oxoimidazolidine (Imid–Cyt)
	5-hydroxymethylcytosine (5-HmCyt)
	5-formylcytosine (5-FoCyt)
	5-carboxycytosine (5-CaCyt)
Guanine	8-hydroxygianine (8-oxoG) 8-oxo-7,8-dihydroguanine (8-oxoGua)
	2,6-diamino-4-hydroxy-5-formamidopyrimidine (Fapy–Gua)
	2,2,4-triamino-5(2H)-oxazolone (oxazolone)
Adenine	8-oxo-7,8-dihydroadenine (8-oxoAde)
	4,6-diamino-5-formamidopyrimidine (Fapy–Ade)
	Inosine

## Theoretical basics of immunoassays

Immunometric methods are based on carrying out specific reactions between the antigen and the antibody. Both, the course and the result of the reaction depend on the structure of these components and the way they are combined.

An *antigen* is a protein, which after its appearance triggers an immune response. The number of antibodies may be influenced by the phylogenetic distance between the antigen donor and the sensitized organism. The more the antigen is “foreign” to the organism (has greater phylogenetic distance), the greater the immune reaction may be. There are cases in which the immune response is small and difficult to detect. To increase the number of antibodies produced, compounds or mixtures, thereof, are used, which are called adjuvants. When administered together with the antigen, they intensify the immune response. Examples of the most commonly used adjuvants are CFA (complete Freud’s adjuvant), IFA (incomplete Freud’s adjuvant), RAS (Ribi adjuvant system) or Titermax, which is one of the new generation adjuvants [16].

The term *antibody* refers to proteins (immunoglobulins) secreted by activated B-lymphocytes (plasma cells) during a humoral immune response that is triggered by the introduction of antigen into the body. Antibodies are directed against a specific antigen and are produced easier for larger and less soluble antigens. The goal of the antibodies is to bind antigens to defend organism against external and intracellular bacteria, viruses, parasites, and toxins.

**Fig. 1:**
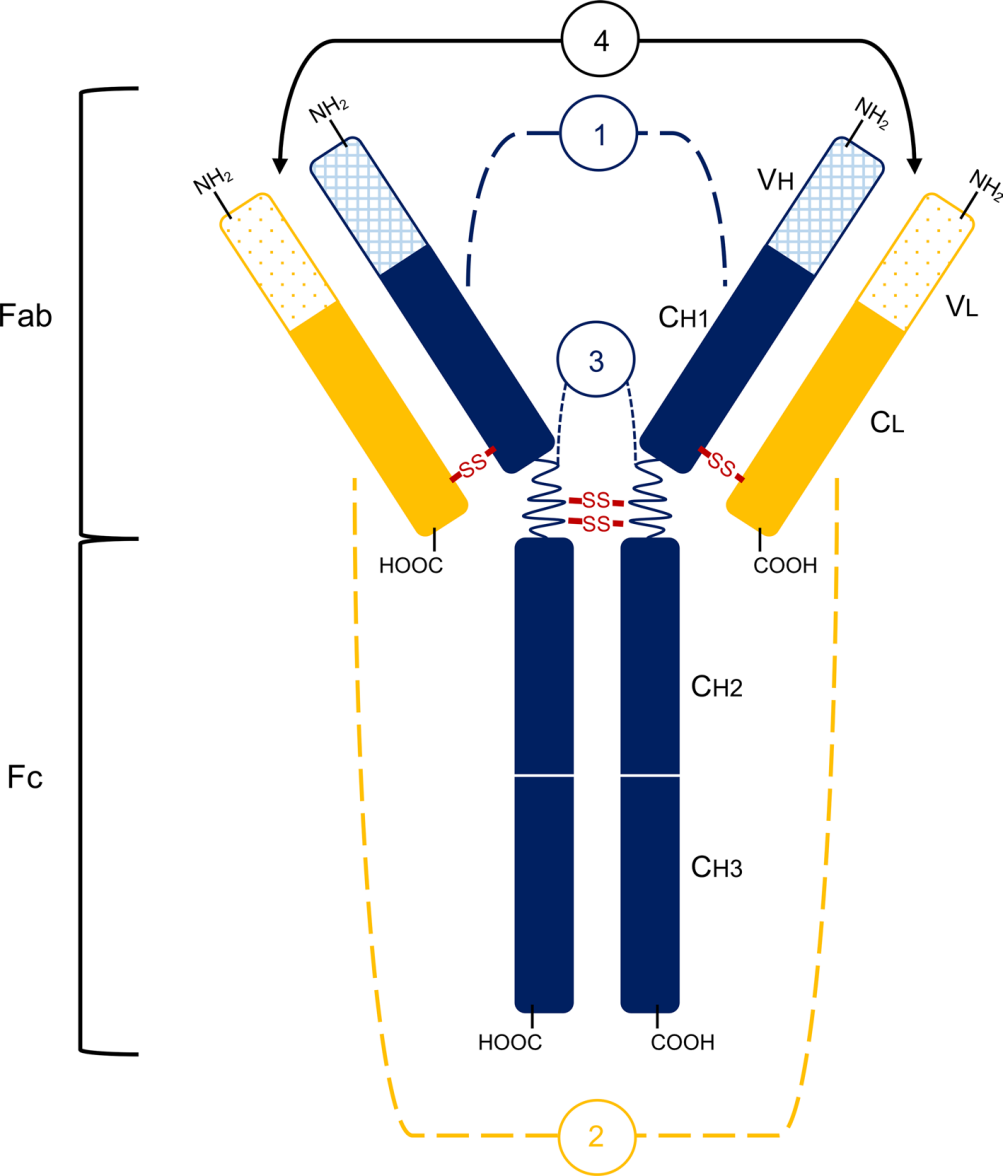
Antibody structure. 1—heavy chain (H), which contains variable domain (VH) and constant domain (from the N-terminus end: CH1, CH2, CH3); 2—light chain (L), which contains variable domain (VL) and constant domain (CL); 3—hinged regions (S–S means disulfide bridges); 4—antigen binding sites [16]

The antibody is composed of four glycoside peptide chains, two of which are referred to as heavy (H) and two are called light (L), with H chains longer than L. There are five types of heavy chains: α (alpha), δ (delta), γ (gamma), ε (epsilon), and μ (mi), which determine the antibody class. Light chains distinguish between types of antibodies and two types of light chains are known: κ (kappa) and λ (lambda). The chains, both H and L, are connected by disulfide bridges in hinge region, which determines the possibility of spreading the arms of the antibody (segmental variation). After hydrolyzation of the antibody, two fragments are formed: Fab and Fc region, whereas after digestion, a F(ab’)_2_ fragment is formed. The Fc region contains only H-chain constant parts, while the Fab fragment includes both, the heavy and variable heavy chain part and the entire light chain. Each of the antibody arms, in the Fab region, has an antigen binding site (paratope) that consists of both types of chain (H and L). Schematic picture of antibody structure and its parts is presented in Fig. 1.

Classes of immunoglobulins, corresponding to the types of chains, are divided into: IgA, IgD, IgG, IgE, and IgM. Each class contains the same types of light chains (κ or λ); however, the type of heavy chains is specific for each class (α, δ, γ, ε, μ). Individual classes of immunoglobulins fulfill different functions in the human body. IgA plays a role in defense mechanisms in the serous and mucous membranes, preventing colonization of pathogens, they are called secreting immunoglobulins as part of saliva and tears. IgD appears on the surface of lymphocytes and acts as a receptor for antigens. IgE plays a major role in allergic reactions, helps to fight parasites, and causes the release of histamine from mast cells. IgG occurs in high concentration in serum and is the main class of immunoglobulins responsible for the organism’s resistance by playing a central role in the defense mechanisms of the cells. This is also the reason why the IgG is mainly acquired for the analytical purposes and is most commonly used in tests such as ELISA. IgG is the only class which is transported from the mother’s body to the fetus, providing immunity up to 3 months of age for the child. Single IgM molecules occur on the surface of B-lymphocytes and serve as a receptor for antigen fusions. IgM are the so-called first-line immunoglobulins; they are the very first developed antibodies during immune response and are responsible for the elimination of pathogens until sufficient amounts of IgG are produced [17].

There are two types of antibodies: polyclonal and monoclonal. Polyclonal antibodies are obtained from sera of sensitized animals and are directed against different epitopes of the antigen. An epitope (an antigenic determinant) is a part of the antigen which a free antibody binds, a B cell or T-cell receptor. The counterpart of an epitope is called a paratope. Monoclonal antibodies are produced by cells obtained by hybridizing spleen cells of an animal sensitized with myeloma cells. These antibodies, unlike polyclonal antibodies, are directed only against one epitope of the antigen. This property results in a much higher specificity of these antibodies but is also associated with a higher price.

The reaction between the antibody and the antigen proceeds according to the equilibrium reaction scheme type A + B → AB. Based on this relationship, two basic concepts are distinguished in immunology: affinity and avidity. *Affinity* means the binding strength of a single antigenic determinant by a single paratope. The affinity value depends on the strength and rate of formation of bonds between the antigen and the antibody. *Avidity* is a term analogous to affinity, but it determines the binding strength of an antigen with many antigenic determinants with antibodies of different specificity. The total energy of such bonds is much higher than the total energy of individual antibodies. The method for determining the average affinity was elaborated by Figuret et al. in 1985 using ELISA technique [18].

The basic types of markers in immunometric methods are radioactive isotopes, fluorochromes, enzymes, and proteins containing heavy metal. Using those types of markers as a criterion, four groups of methods can be characterized: radioimmunoassays (RIA), immunofluorescent assays (IMF), immunoenzymatic assays, and assays with heavy metal-labeled antibodies.

*Radioimmunoassays (RIA)* are classic quantitative methods that use radioactive isotopes for detection of antigen–antibody interactions through direct or indirect measurement of the unlabeled macromolecular substance binding to a specific receptor system (e.g., antibody) [19]. The higher the radioactivity of the sample, the higher the concentration of the substance of interest should be detected. There is a number of isotopes available; in most cases, ^125^I iodine, ^32^P phosphor, ^14^C radiocarbon, and ^3^H tritium are considered. Depending on the chosen compound, a different type of radiation is emitted: β particles (low penetration power) or γ rays (high penetration power) [2, 20, 21]. Equipment such as gamma counter measures both, β particles and γ rays, with a better result observed for the second ones. No need for sample preparation and a short time of analysis are advantages of using such counters. In spite of being one of the first techniques in the field, RIA is still employed in many diagnostic tests due to its sensitivity and simplicity. It consists of three stages: immunoreaction (antigen/antibody binding), competitive binding or competitive displacement reaction, which gives specificity and measurement of radio emission providing high sensitivity (0.0006–0.006 µg antibody/ml) [22]. Moreover, RIA is cheap in operation; however, specialized instruments, meeting legal terms, and training of personnel are required prior to launching experiments. For years, the technique was applied for diagnostic purposes, mainly to analyze a chosen antibody or antigen to diagnose patient’s disease [23, 24]. Nowadays, it is also used in screening for the immunity, allergens in the food industry or in the molecular biology laboratories. In the context of DNA damage detection, this method allows to effectively determine 6–4 photoproducts and cyclobutene dimers in DNA in variety of samples: cell cultures, organ cultures, tissues (e.g., from biopsies), bone marrow cells, urine, and others [25]. Few variants of the method are known which broaden range of applications: IRMA (immunoradiometric assay), RAST (radioallergosorbent assay), and Farr assay which is employed for detection of anti-double stranded DNA (anti-dsDNA) antibodies and characteristics of systemic lupus erythematosus (SLE). In this method, anti-dsDNA/DNA complexes are separated from free DNA labeled with radioisotopes through ammonium sulfate precipitation after which dissociation of complexes takes place [26–30].

*Immunofluorescent assays (IMF)* adopt antibodies or fluorochrome-labeled antigens, where the result of the reaction, in the form of colored complexes, can be read, e.g., in ultraviolet light. Fluorochromes are a group of organic dyes capable of emitting light due to the excitation of a suitable radiation source. The emission of light within the visible light range is necessary for the fluorochrome to be employed in the analysis. The most popular fluorochromes are: green fluorescein isothiocyanate (FITC), red tetramethylrhodamine (TRITC), red sulforhodamine 101 acid chloride (Texas red^®^, TR), and orange R-phycoerythrin (PE) [31].

Enzymes which represent *immunoenzymatic assays* are most commonly used as a label. The method is indirect—to determine the number of antibodies in a complex, the activity of the enzyme should be detected. Enzymes are attached to the antibody or antigen by means of glutaraldehyde or sodium periodate or enzyme–antienzyme complex (e.g., peroxidase–antiperoxidase (PAP), alkaline phosphatase–alkaline antiphosphatase (APAAP)). For the enzymatic reaction to occur, the presence of substrates is necessary—hydrogen peroxide and the appropriate chromogen in which the reduction reaction form a water molecule of hydrogen peroxide and a colored compound of a chromogen. The complexes obtained differ in color depending on the enzyme and the chromogen introduced to the reaction. The most common enzymes are summarized in Table 2.

**Table 2:** Examples of enzymes used as markers in immunoassays with indication of possible chromogens and colors of complexes forming in the reaction [16]

Enzyme	Chromogens	Color
Horseradish peroxidase (HRP)	orthophenylenediamine (OPD)	Orange
	3,3-diaminobenzidine (DAB)	Brown
	3-amino-9-ethylcarbazole	Red
	tetramethylbenzidine (TMB)	Yellow
	dimethylformamide (AEC)	Pink
	2,2′-azino-bis(3-ethylbenzothiazoline-6-sulfonic acid) (ABTS)	Green
Alkaline phosphatase (AP)	naphthol phosphate AS-MX + Fast blue BB	Blue
	naphthol phosphate AS-TR + hexazonium fuchsine	Red
	5-bromo-4-chloro-3-indolyl phosphate + nitrotetrazolium blue	Indigo
β-D-galactosidase	6-bromo-2-naphthyl + Fast blue BB	Blue–purple
	5-bromo-4-chloro indolyl + Fast blue BB	Blue–green
Glucose oxidase	Phenazine methosulphate + nitrotetrazolium blue	Blue–purple

A different example of ligand labeling is labeling with colloidal gold. This process is based on specific physicochemical properties of gold that enable non-specific adsorption of chemical compounds on the surface of gold molecules. The classic strategy in the context of DNA studies is to label single-stranded DNA (ssDNA) with gold nanoparticles (AuNPs) and observe the change in color during the hybridization reaction of DNA strands. Tests using this type of mechanism are easy to conduct which is beneficial for researcher. Colloidal gold was found useful in immunoassays, ELISA in particular, where it can be applied to the whole range of detected compounds and biomolecules [32, 33]. Currently, gold nanoparticles may also find its application as a probe for detection of DNA damage with QDs (quantum dots). AuNPs bind to ssDNA forming a platform for hybridization of the second strand containing mutation of interest which thereafter is detected by quenching of QDs after it binds to AuNPs–dsDNA [34].

Immunometric methods find many applications in diverse scientific fields, e.g., agriculture, environmental protection, veterinary science, medicine, food analysis, and molecular biology. Applications range from the analysis of compounds such as proteins and peptides, microorganisms (molds, bacteria), toxins (mold, bacterial, and other), hormones, antibiotics, vitamins, pesticides, and metal ions to small particles such as nucleotides, DNA or RNA [35–37].

## Enzyme-linked immunosorbent assay (ELISA)

**Fig. 2:**
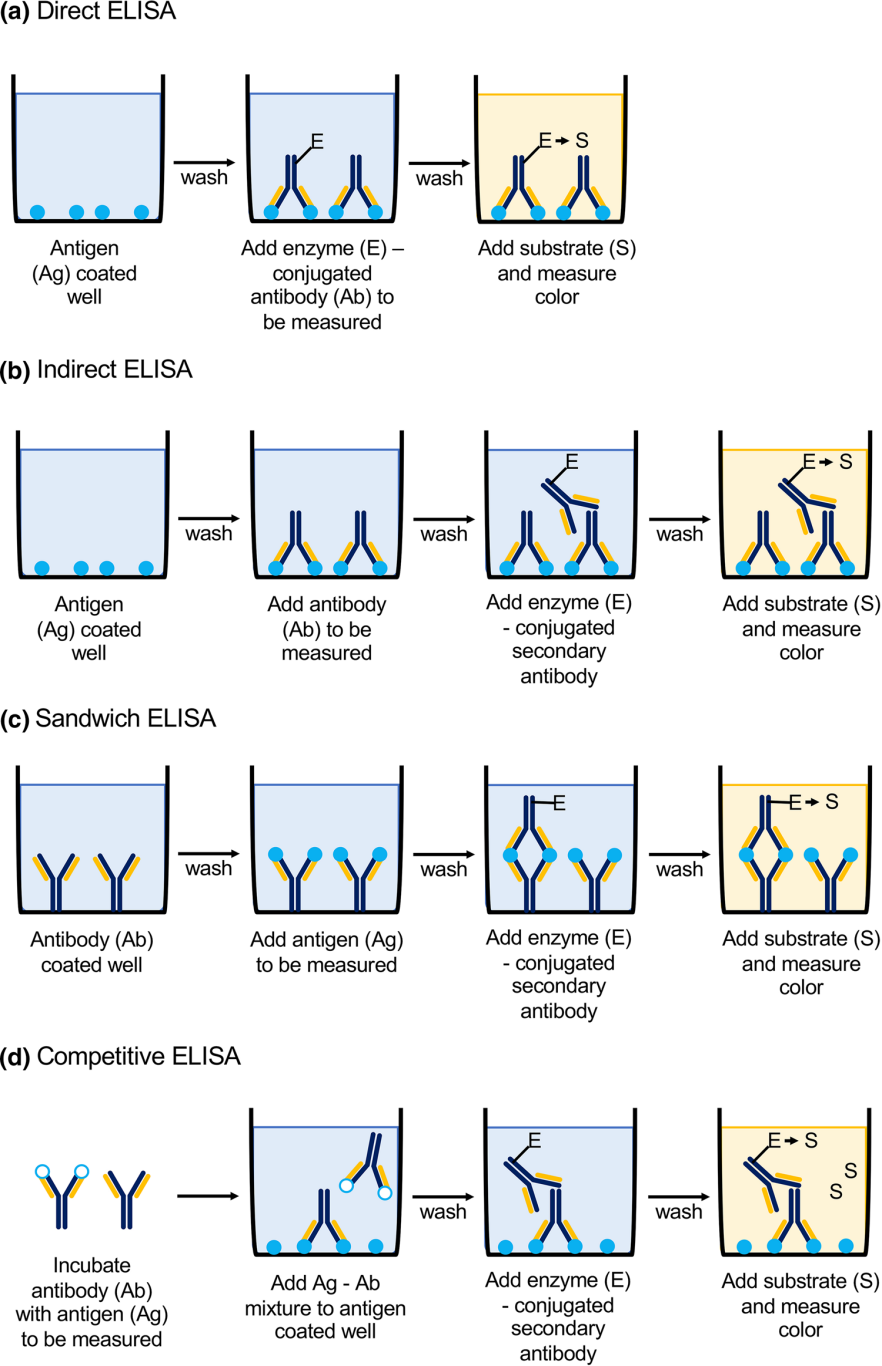
Schematic presentation of basic types of ELISA (enzyme-linked immunosorbent assay): **a** direct, **b** indirect, **c** sandwich, **d** competitive; *Ag* antigen, *Ab* antibody, *E* enzyme, *S* substrate [42]

*ELISA* belongs to the group of solid-phase tests and is an enzyme immunoassay that uses a color reaction of the enzyme coupled to the antibody. It is performed on microplates, where the adsorption of antigens or antibodies takes place on the walls of the wells (depending on the chosen technique). The technique is based on the immobilization of the antigen on the surface of the solid phase and the introduction of biological material that contains antibodies specific for the antigen, covalently linked to the enzyme [38]. The antigen forms an immune complex with the antibody, thanks to which the antibody is also bound to the substrate. After introducing the substrate, the enzyme catalyzes the reaction, which results in a product, usually a colored one. The concentration of this product can be determined spectrophotometrically allowing quantitative and qualitative analysis. The amount of product formed corresponds to the concentration of the antibody–antigen complex which is calculated based on a standard curve determined using specific standards. The quantity of a product, equivalent to the concentration of the antibody–antigen complex, is proportional or inversely proportional (depending on the type of test) to the quantity of the given substance in the sample. There are several types of ELISA test—based on direct reaction (non-competitive, competitive with catching antibodies, competitive with catching antigen), indirect reaction (normal, with the avidin–biotin system, with the enzyme–antienzyme complex, e.g., PAP), “sandwich”-type test and competitive or non-competitive reaction [39–41]. Basic types of ELISA are presented in Fig. 2.

In laboratory diagnostics, an indirect ELISA is most commonly employed. Two types of antibodies are used: primary, which recognizes a given antigen and frequently is a monoclonal one, so that the designation is as specific as possible and secondary, which is labeled and recognizes primary antibody. The primary antibody is not labeled; therefore, costs of analysis are significantly reduced, which is beneficial, given the high price of monoclonal antibodies. A secondary antibody is labeled and attaches to a given primary antibody. The standard ELISA technique does not show high sensitivity and, therefore, it was refined by creating “sandwich”-type ELISA, which differs in that the antigen is not immobilized directly on the substrate but is bound to the coat antibody introduced on the microplate first. This assay is used, among others, to detect the amount of the test protein in a sample and in diagnostic tests, e.g., to determine the titers of antibodies in the blood. Detection of DNA oxidative damage, e.g., thymine glycols or 8-hydroxyguanine is also possible with commercially available kits [5, 43].

The ELISA test and its many variants are one of the most popular and common techniques due to a number of advantages such as versatility, high sensitivity (depending on the method), selectivity, specificity of reactions, the possibility of multiple repetitions, relatively short analysis time, simplicity of execution, and low cost, which makes the technique profitable in both practical and economical terms. However, there are also some disadvantages: a limited number of commercially available specific antibodies and high cost of purchase, reduced specificity of antibodies labeled with enzymes, and the need to obtain appropriate dilutions of reagents in case of indirect techniques. Moreover, in case of DNA damage detection, the possible crossreactivity of applied antibodies with DNA bases is major drawback. All these shortcomings have led to the development of improvements to this method and other more efficient techniques have been introduced.

## Detection enhancement approaches

### Tyramide signal amplification (TSA)

*TSA* is an amplification approach that uses biotinylated tyramine to increase the detection sensitivity of a target protein or nucleic acid. This method can also be found in the literature under the name ImmunoMax or CARD (catalyzed reporter deposition) [44]. The TSA reaction is based on the ABC method (avidin–biotin–peroxidase complex). Biotinylating of antibodies and markers such as peroxidase takes place, while avidin acts as a bridge connecting biotins that are part of these proteins. The affinity of the avidin and biotin molecules is high, whereby the bonds are stable.

**Fig. 3:**
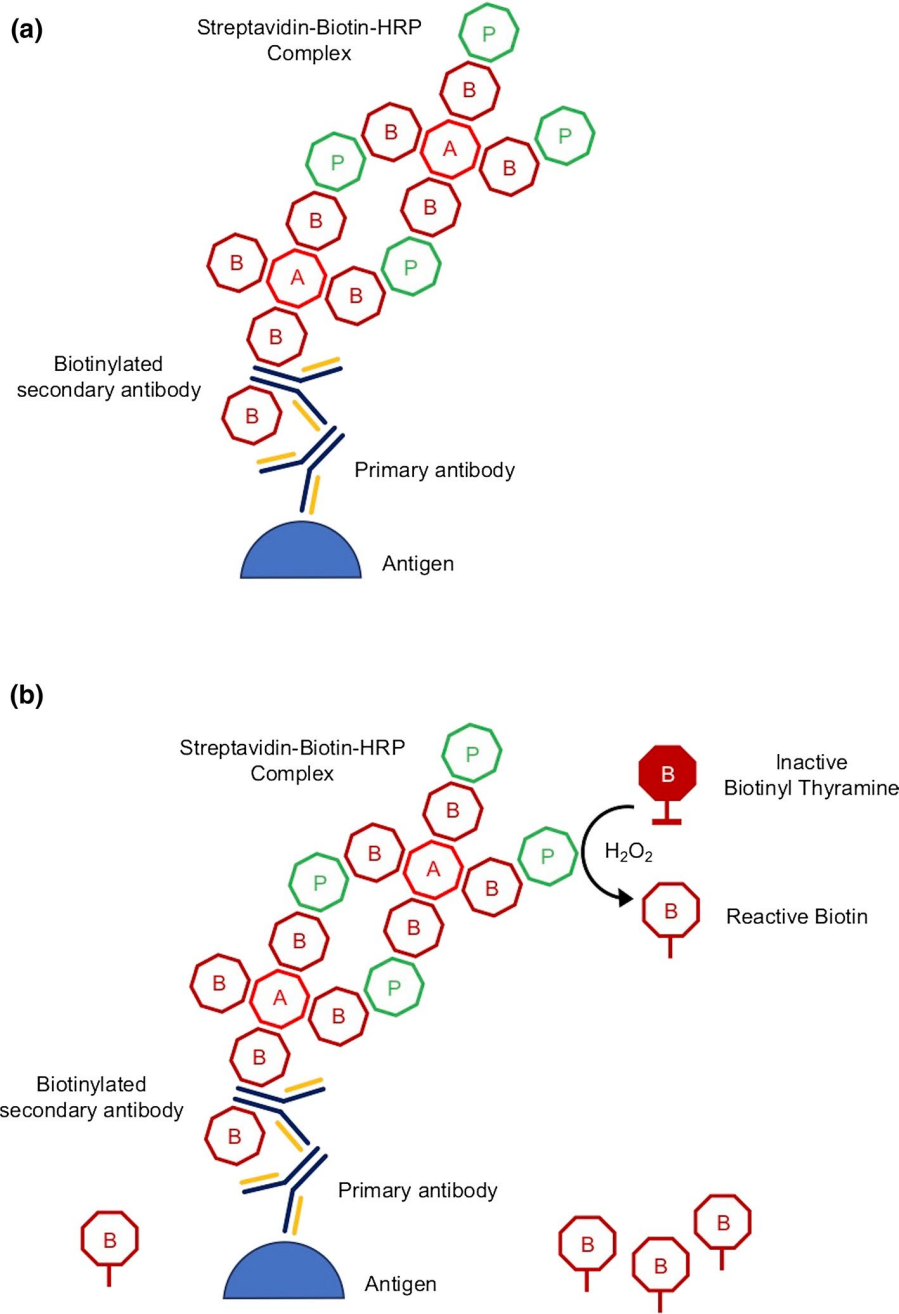
Schematic overview of the **a** standard ABC and **b** biotinylated tyramine amplification (TSA) method; *A* avidin, *B* biotin, *P* enzyme (*HRP* horseradish peroxidase) [44]

The reaction of TSA using biotinylated tyramine is initially performed like the classic ABC reaction (Fig. 3). However, after the peroxidase introduction, biotinylated tyramine is used to build up additional biotin molecules at the site of the reaction between the antigen and the antibody. The goal is to obtain as many molecules of biotin as possible in the solution while introducing to the reaction only one enzyme molecule (peroxidase). Biotin present in the reaction environment can be detected by many methods, e.g., the avidin–enzyme complex or streptavidin-biotinylated enzyme may be adopted. The advantage of the TSA method is its sensitivity—antibodies can be used in a 10–100 times lower concentration compared to indirect ELISA reaction such as PAP method (peroxidase–antiperoxidase) [45].

TSA can be applied in FISH and, due to its sensitivity and speed, it allows to detect short oligonucleotide probes often within a single day of analysis, which is not possible with classic protocol. Moreover, this method needs 2–10-fold lower concentrations of hybridization probes and more than one probe can be used in reaction, thus allowing multiple target detection [44, 46].

### Enhanced polymer one-step staining (EPOS)

**Fig. 4:**
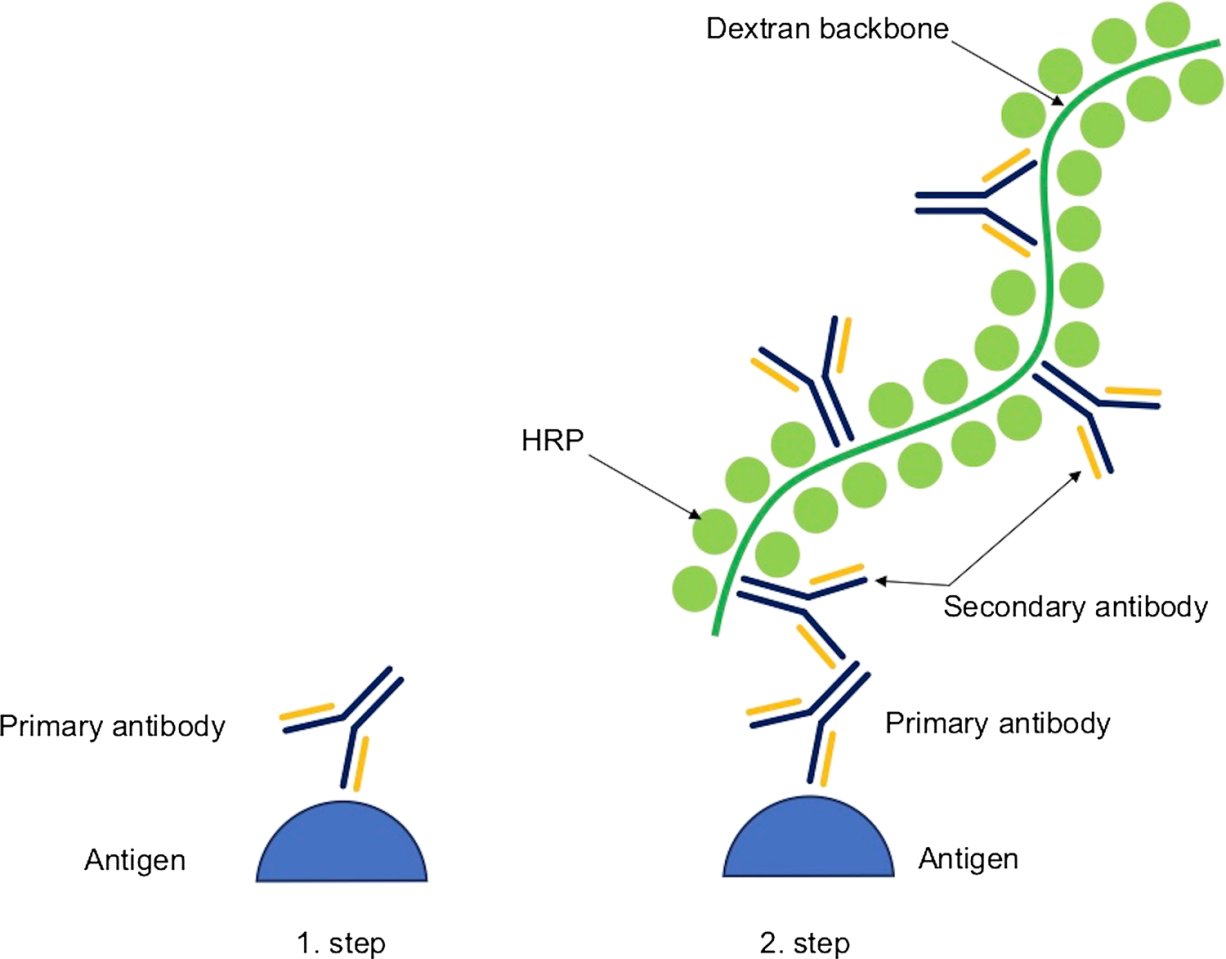
Schematic overview of the EnVision method. In the first step, primary antibody binds to antigen, and in the second step, complex of enzyme and secondary antibodies attached to dextran backbone is introduced to the reaction; *HRP* horseradish peroxidase [47]

The *EPOS* method has been developed to simplify and shorten the time of performing immunochemical reactions and to eliminate or reduce unspecific staining resulting from biotin activity. However, sensitivity remains close to the sensitivity of the ABC reaction. The EPOS method starts with the introduction of a ready complex into the reaction environment containing a large number of antibodies and a marker (e.g., HRP) connected to each other by a branched, inert polymer, e.g., dextran which dissolves in water, making the entire complex well soluble and stable [47]. A maximum of 70 molecules of the enzyme and 10 molecules of the secondary antibody can be attached to a single polymeric backbone. To improve this standard protocol, the EnVision system has been developed (Fig. 4). The system is based on a two-step reaction, where in the first stage, the primary antibody is introduced, and in the second stage, a complex of secondary antibody, enzymes (e.g., HRP), and polymer is introduced.

In this variant of the EPOS method, the total incubation time is shorter and larger variety of primary antibodies can be applied, which increases the number of possible applications of the method. Moreover, in comparison to the one-step EPOS, the two-step EnVision technique showed higher sensitivity—it is possible to use greater dilutions of primary antibody solutions without losing the accuracy of the results [48, 49]. The EnVision system is constantly improved by its manufacturer. An example is EnVision DuoFLEX +, which allows the introduction of a mixture of secondary antibodies to the reaction, each of which is directed against a different primary antibody. This allows simultaneous execution of two measurements, which significantly saves time and gives the possibility of a reliable comparison of reaction results [50–52]. The next generation of techniques based on polymer complexes is the PowerVision detection system. It uses small, multifunctional polymeric connectors for the better tissue permeability than dextran and, at the same time, shows higher detection sensitivity [53].

Due to their sensitivity, the mentioned systems have found their application in the study of small molecules such as antibody–antigen interactions. In in vitro diagnostic tests, EPOS/EnVision may be applied to assess progression and malignancy of the tumor in cancer patients through detection of PCNA (proliferating cell nuclear antigen) and Ki-67 antigen in the frozen sections of tissues [48]. Moreover, the method can be used in case of infectious disease such as HIV and tuberculosis for detection of DNA targets, which is possible at the level of fM (10^−15^). Still, signal amplification techniques may also be applied, e.g., quantum dots, which yet increase the levels of detection [53].

### Time resolved amplified cryptate emission (TRACE)

**Fig. 5:**
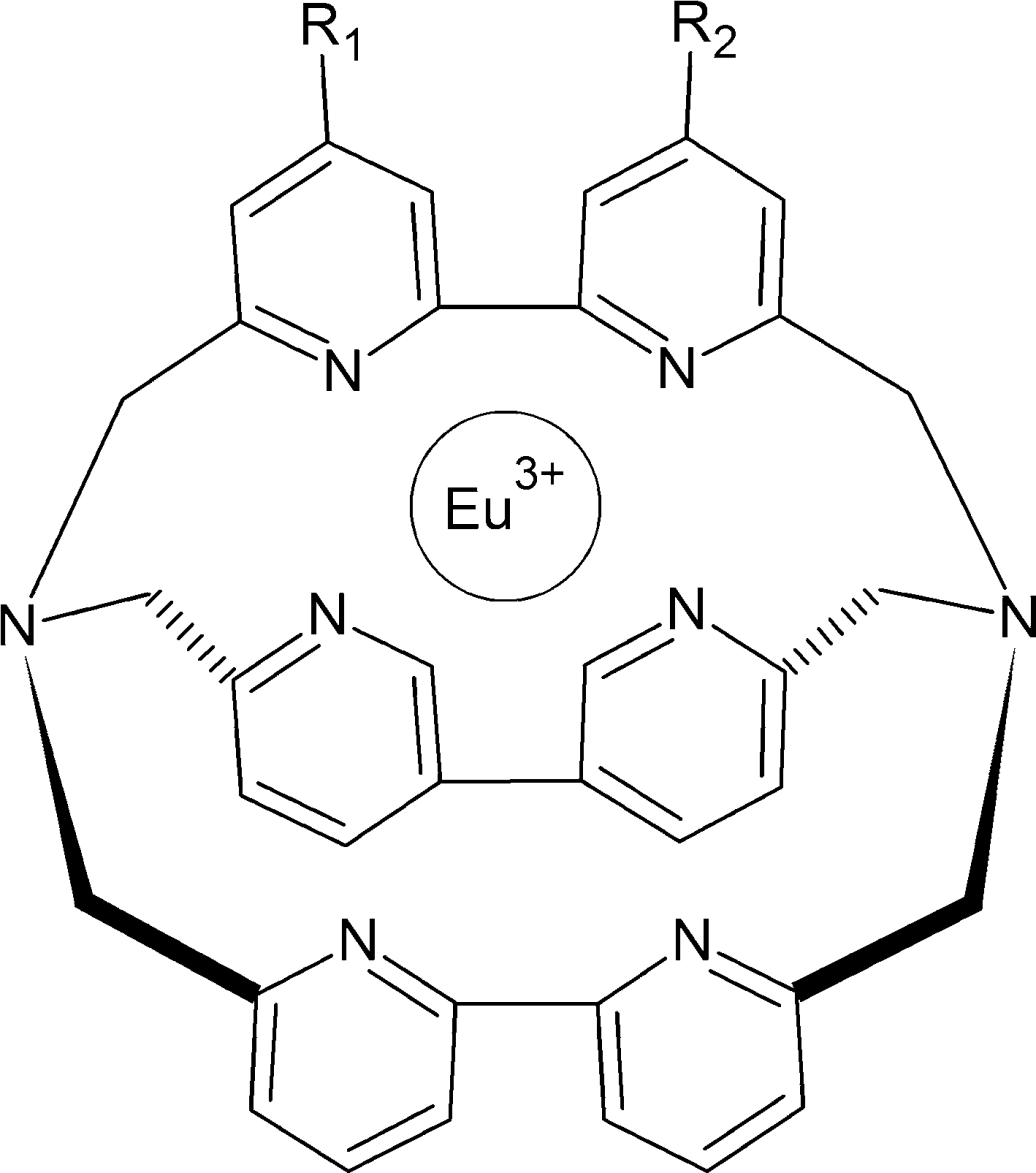
Chemical structure of Europium cryptate

**Fig. 6:**
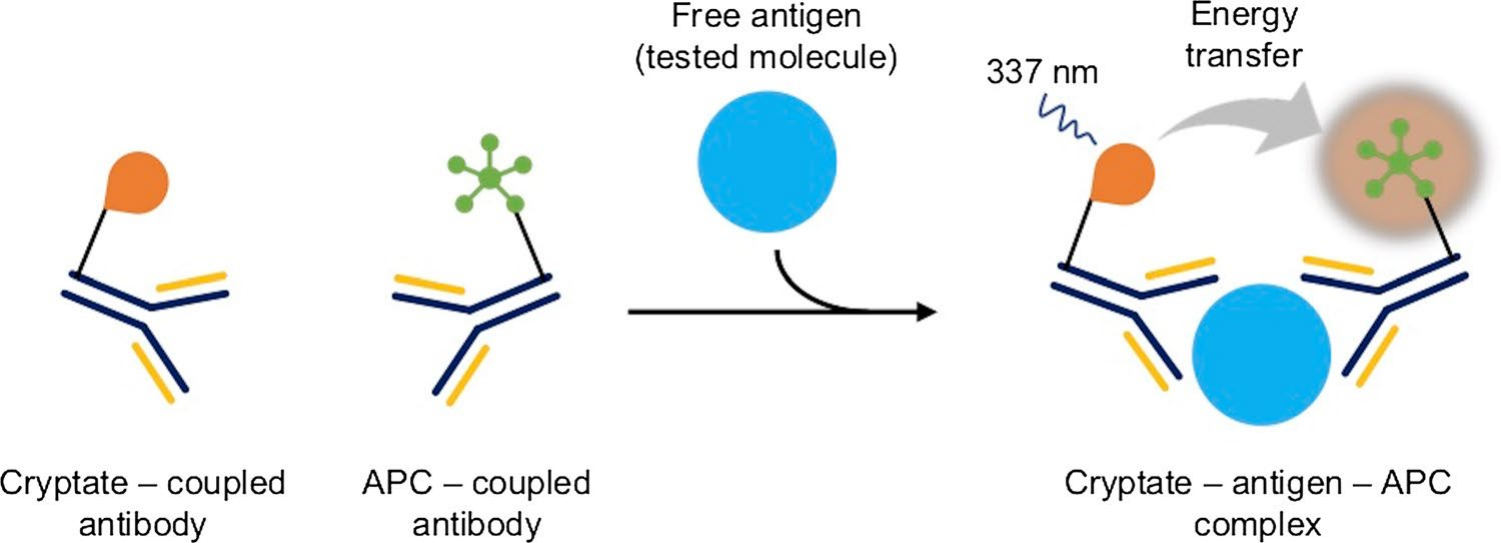
Schematic overview of TRACE reaction. Europium cryptate serves as an energy donor and APC (allophycocyanin, XL665) serves as an energy acceptor. When donor and acceptor are bound to antigen (tested molecule), upon excitation at 337 nm, cryptate transfers energy to APC. Signal is measured at 665 nm and corresponds to the quantity of bound cryptate and fluorophore. It is proportional to analyte concentration [54]

The TRACE technique is based on the phenomenon of non-radioactive fluorescence energy transfer via resonance between a donor and an acceptor. The donor is an Europium cryptate (Eu) or Terb (Tb) (Fig. 5) bound to an antibody, and the acceptor is a fluorophore, allophycocyanin (APC, XL665) bound to a different antibody. The energy is transmitted when both complexes bind to the molecule being tested (e.g., antigen during the complete immune response) and the donor molecule is excited at 337 nm [54, 55]. The reaction is schematically presented in Fig. 6. The emission range of donor and acceptor fluorescence differs significantly from one another—cryptate gives signal at 620 nm and APC at 665 nm. In addition, Europium cryptate is characterized by a long fluorescence life (10^−6^ s) as opposed to APC (10^−9^ s). Nevertheless, after transfer of energy from the donor to APC, it exhibits a fluorescence life estimated in microseconds. These two parameters (spectrum and time of illumination) allow to clearly distinguish the related particles from free particles in the solution. The measured fluorescence value is proportional to the concentration of the analyte determined.

The TRACE technique allows one-step measurement in a homogeneous solution. The results obtained with this method are highly reproducible and precise, and the short incubation time (from 9 to 59 min, depending on the chosen type of test) facilitates work. The TRACE method has been adapted to work with KRYPTOR systems. This system may be used, e.g., for follow-up testing of cancer patients through detecting level of marker proteins, e.g., chromogranin A. It is employed also in thyroid diagnostics through detection of anti-Tg_n_ (thyroglobulin) and anti-TPO_n_ (thyroid peroxidase) antibodies. In the context of molecular interactions, nucleotides labeled with Europium cryptate serve as the new type of biotinylated labels for the RNA:DNA hybrid detection through measurement of FRET (fluorescence resonance energy transfer) signal. The FRET technique allows to examine structure and dynamics of macromolecule interactions such as proteins and its approaches in the range of 10–100 Å, in many solution conditions which is greatly beneficial for nucleic acid studies [56–60]. For the purpose of nucleic acid analysis (DNA hybridization and sequencing, PCR), the OLA (oligonucleotide ligation assay) method is known to be of a great use. The OLA is appropriate for the detection of specific mutations in nucleotide sequence, it incorporates cryptate label tris-bipyridine-Eu(3 +) thanks to its long-lived fluorescence which allows to reach high sensitivity through time-resolved method of detection. TRACE in combination with OLA allows detection of single nucleotide. An example of OLA–TRACE application was demonstrated in the case of K-ras oncogene mutation analysis [61].

### Surface plasmon resonance (SPR)

The SPR phenomenon is one of the optical phenomena that have their application in chemo- or biosensors. Such sensors consist of a receptor layer that interacts with a given substance and a component that converts the measured parameter into a signal (optical, acoustic, electric). In the case of biosensor, the receptor layer is formed by a biological material (e.g., enzymes, antibodies, microorganisms, tissues, nucleic acids or other biologically active compounds) [62]. The biological component remains in direct contact with the sensor which interacts with the substance of interest. It gives a signal strictly dependent on the concentration of the analyte in the test sample.

Due to their sensitivity and selectivity, biosensors allow to measure the analyte in a simple and quick manner; therefore, they have a wide range of application, e.g., in environmental monitoring, clinical diagnosis, pharmaceutical science, and food research [63]. Worth mentioning are also DNA-based biosensors which rely on the DNA hybridization process and occur as solid-phase-based reactions [64]. Furthermore, biosensors are used to detect DNA damage. For example, damage induced with hydroxyl radicals may be determined by the electrochemical DNA biosensor with carbon electrode and dsDNA adsorbed on its surface as a method of identifying genotoxic chemicals [65]. Different study shows an impedimetric biosensor which allows to detect DNA damage (also induced with hydroxyl radicals) through implementing gold electrode with DNA and gold nanoparticles as a receptor layer as a method for testing protective/antioxidative properties of deferoxamine (DFO) [66].

**Fig. 7:**
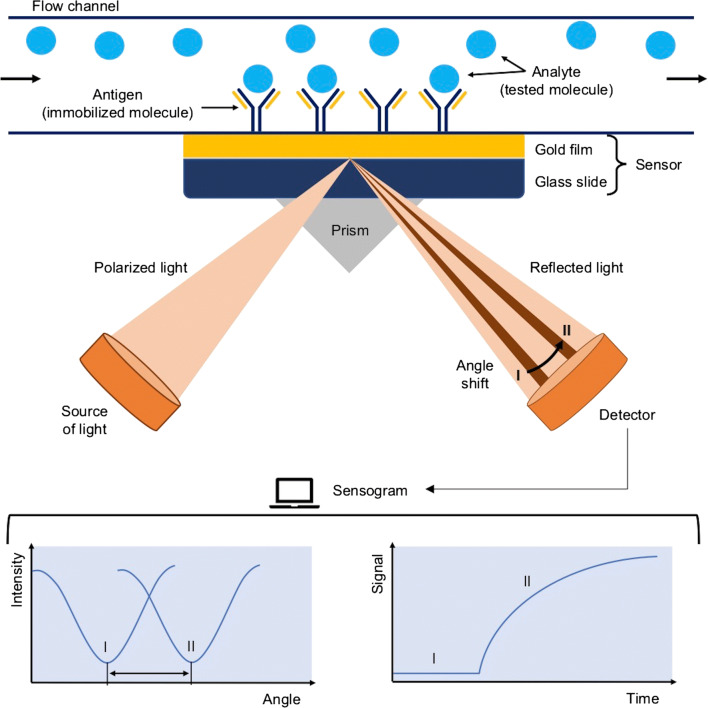
The scheme of operation of surface plasmon resonance (SPR) [68]

Analytical instruments operating on the basis of surface plasmon resonance use the phenomenon of optical measurement of the refractive index on the surface of the sensor (which usually is coated with metal, with ligand molecules immobilized on its surface). The sensor forms the lower wall of the flow chamber, through which a properly selected buffer flows. Analyte is injected into the buffer and as it binds to the ligand, the molecules accumulate on the sensor surface leading to changes in the refractive index and reflected light, which is proportional to the mass of accumulated molecules. The scheme of the SPR method is shown in Fig. 7. The detected signal is read in the RU (resonance units), where 1 RU corresponds to approximately 1 pg/mm^2^ of the tested substance [67]. Results (in a form of a sensogram) contain information whether the interaction takes place, what is its power and specificity, how much of the analyte has been bound on the surface of the sensor, and what is the kinetics of the interaction.

The SPR technique is relatively new and one of the first recommended applications was structure assessment of the recombinant macromolecules through determination of the compatibility of the obtained structure with its naturally occurring form. It has been achieved by comparing the way of natural ligands or monoclonal antibodies attachment. At the moment, multitude of analysis can be performed using the SPR method. It is especially adapted for the small molecules as they can be determined with very high accuracy reaching even 10^−13^–10^−16^ M. Other examples of SPR application are: quantitative and qualitative determination of toxins, monitoring gene expression, study of interactions between antigen and antibody, DNA–protein, RNA–protein, DNA–DNA, RNA–RNA, cell–protein, and kinetics of those reactions [69, 70]. The method has a number of advantages, such as very high sensitivity and selectivity of the measurement, the ability to study the kinetics of the reaction in real time, the lack of complex sample processing, short analysis time, and no need of using markers or labels. In addition, the sensor can be used repeatedly after appropriate regeneration [71].

In the context of DNA studies, SPR is employed for DNA kinetics determination, DNA strand separation and hybridization, enzymatic mechanisms, and even for detection of single-point mutations [72]. Studies show that the DNA-based SPR biosensor is able to analyze DNA sequences and detect differences of one nucleobase. In this case, mutation at 248 codon in DNA extracted from the wild-type and mutated cancer cell line with *TP53* gene mutation was determined by a gold surface sensor coupled with synthetic oligonucleotide probes [73]. Additionally, surface plasmon resonance is useful in DNA damage research as a real-time analysis method of binding affinity of oligonucleotides and enzymes. Deutsch et al. analyzed the interaction of DNA templates with 8-oxoG (7,8-dihydro-8-oxoguanine) in its sequence with hS3 (human ribosomal protein S3) which is involved in cleaving AP sites (apurinic/apyrimidinic sites) and protein translation. Thanks to SPR technique, the research showed exact binding affinity of hS3 to 8-oxoG which was 3–5-fold higher than binding affinity of OGG1 (8-oxoguanine DNA glycosylase) indicating that hS3 protein may have its functions in the DNA base excision repair system (BER) [74].

Currently, there is a spectrum of devices available—one among many solutions is the FLEXChip system, which is one of the large format flow cell SPR array-based systems and allows monitoring of approximately 400 reactions simultaneously by a camera that reads the reflected light rays for each molecule to be measured [75]. On the other hand, DNA-based SPR biosensors have hardly been used with biological material. New combinations of SPR, sensors (e.g., mass spectrometry), and matrices (e.g., aptamers, DNAzymes) are developed to allow analysis of complex biological systems which is a promising prospect for the future of this method [71].

### Quantum dots (QDs)

**Fig. 8:**
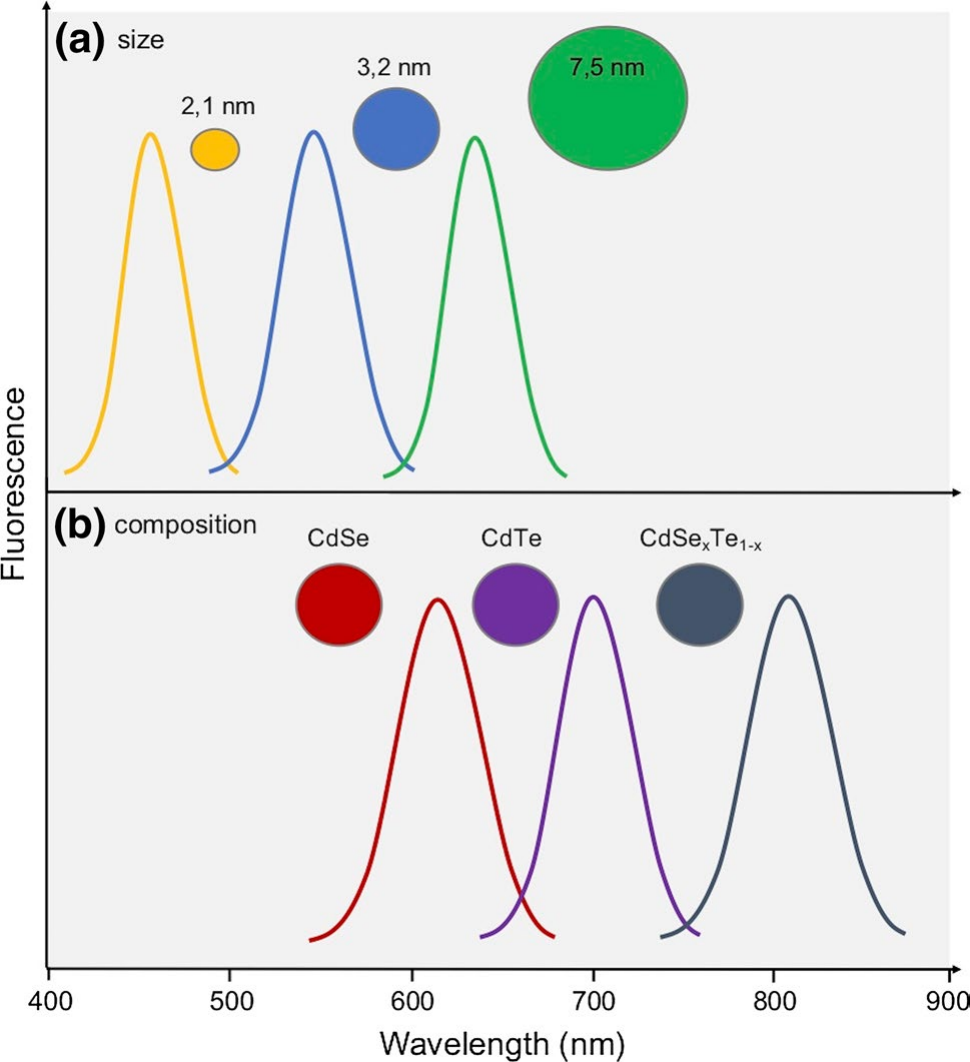
Change in fluorescence emission band in relation to **a** crystal size or **b** chemical composition of quantum dots [77]

Quantum dots are semiconductor crystals of small size, the core of which is composed of elements of groups IIB–VIA of the periodic table, e.g., CdSn. The coating consists of elements belonging to groups IIIA–VA, e.g., InP. Dimensions of quantum dots lie within the limit of 10–20 nm [76]. The physical properties (melting point, absorption, emission of radiation, electrical conductivity) depend not only on the structure and chemical composition of a given crystal, but also on its dimensions. By manipulating QDs diameter in the range of 2–7.5 nm, the emission of any wavelength in the entire visible spectrum (450–650 nm) can be obtained (Fig. 8a). For QDs at constant diameter, changing only one of the compounds in the dot’s composition allows to obtain fluorescence emission in the range of 610–800 nm (Fig. 8b) [77].

Quantum dots, thanks to their properties, (narrow emission bands, high photostability, high emission efficiency, small size) enable to study the behavior of individual particles marked with single dots. In combination with existing methods such as ELISA, PCR, FRET, FISH or Western blot, they improve detection levels. In the ELISA test, QDs are used for labeling antibodies successfully replacing fluorophores, thus, increasing sensitivity and speed of analysis [78, 79]. When compared to fluorescein isothiocyanate and rhodamine 6G, QDs show a broader excitation profile, the emission band is narrower and more symmetrical than for both fluorophores, which allows to obtain highly specific results [77, 80]. The comparison of the QDs and fluorophores’ main properties is summarized in Table 3.

**Table 3:** The comparison of general properties of quantum dots and fluorophores [77, 78]

	Quantum dots	Fluorophores
Excitation	Wide range (UV light can excite a dot of any size)	Small range
Emission range	20–40 nm	50–100 nm
Fluorescence life	10–40 ns	<10 ns
Photostability	Over 14 h	20 min (fluorescein)
Molar extinction coefficient	10^5^–10^6^ [1/M*cm]	10^3^–10^5^ [1/M*cm]

In the case of PCR, QDs can increase specificity of amplification through interacting with Taq polymerase or DNA templates, which improve the specificity [78]. FRET technique combined with QDs creates a new platform for DNA detection and with enhanced efficiency of analysis. Different combinations of QDs and fluorescent dyes were demonstrated for a different purpose: DNA detection (QDs/Cy5 (cyanine)), nucleic acid detection (CdSe/ZnS-QDs/TAMRA (carboxytetramethylrhodamine)), and detection of DNA hybridization (TGA (thioglycolic acid)-CdTe-QDs/Cy3) [78, 80, 81]. FISH can incorporate oligonucleotide probes labeled with QDs. By labeling linear DNA molecule with biotin and digoxigenin, two-color determination of the orientation of a single DNA molecule is achievable. Once labeled with QDs, DNA molecules are detected with fluorescence microscopy [82, 83]. DNA damage and quantification of nucleic acids may be achieved through graphene quantum dots binding to damaged ssDNA pre-labeled with gold nanoparticles [34]. Recently, CdTe/ZnSe QDs have been proved to interact with nucleobases (which show different fluorescent emission for every base), allowing to apply this method to detect DNA damage and mutations at the level of 500 pM of DNA [84]. A different study shows the possibility of detection of DNA damage caused by UV radiation and hydroxyl radicals through binding carbon dots (C-dots) to the genomic DNA extracted from PC3 cells [85]. Wang et al. described an efficient method of detection of BPDE (anti-benzo(a)pyrene diol epoxide)–DNA adducts using a QD–antibody–DNA complex as an analysis target. It allowed to measure adducts’ concentration at the level of 10^−15^ M, which is 5400 times more sensitive than ^32^P-labeling. According to the authors, this method may also be employed for DNA damage and repair studies [86].

## Summary

Spontaneous and environmentally induced DNA damage affects all living cells. Detection of this damage and exploring DNA repair systems are important; hence, more attention is paid to the innovation and development of highly precise techniques. On the other hand, classic methodologies such as PCR cannot recognize type of detected DNA damage, other techniques give chance to detect single cells with DNA damage in a given population (comet assay), analyze the ratio of apoptotic to necrotic cells (FCM and FISH) or detect oxidative damage, and quantify thymine dimers (HPLC–electrospray tandem mass spectrometry and GC–MS) [5, 87–89]. Methodology is crucial for sensitive detection of genome DNA damage, characterization of damage type, and quantification of DNA damage and repair mechanisms. Possible matches between methods and DNA damage are presented in Table 4.

**Table 4:** Chosen types of DNA damage, antibody, and method recommended for detection with possible detection level [90–95]

DNA damage	Antibody/method	Level of detection
5-HmCyt	Anti-5HmCyt antibody/SPR	1,6*10^−10^–5*10^−9^ M
5-MCyt	Anti-5MCyt monoclonal antibody + HRP secondary antibody/ELISA	4,2*10^−13^ M
8-oxoG	Peroxidase-labeled anti-8-oxoG monoclonal antibody/ELISA	5*10^−12^ g/ml
6–4PP	Anti-6–4PP antibody + HRP secondary antibody/ELISA	n/a
CPD	Anti-CPD antibody + HRP secondary antibody/ELISA	n/a
BPDE–DNA adducts	Anti-BPDE antibody + HRP secondary antibody/ELISA	1,2*10^−10^ M

There is a multitude of known and applied methods in the field of molecular studies of DNA and its damage. From classic to the newest variants of each method, all the advantages and limitations should be taken into consideration while choosing methods for one’s own research. The field of modern techniques is worth exploring, as our knowledge of the world grows, accuracy and precision with which we want to discover the world also grow, provoking further improvement of the tools we need and want to use.
